# The *de novo* Transcriptome and Its Analysis in the Worldwide Vegetable Pest, *Delia antiqua* (Diptera: Anthomyiidae)

**DOI:** 10.1534/g3.113.009779

**Published:** 2014-03-10

**Authors:** Yu-Juan Zhang, Youjin Hao, Fengling Si, Shuang Ren, Ganyu Hu, Li Shen, Bin Chen

**Affiliations:** Institute of Entomology and Molecular Biology, College of Life Sciences, Chongqing Normal University, Chongqing, People’s Republic of China

**Keywords:** onion maggot, high-throughput RNA sequencing, *de novo* assembly, codon usage bias, simple sequence repeat

## Abstract

The onion maggot *Delia antiqua* is a major insect pest of cultivated vegetables, especially the onion, and a good model to investigate the molecular mechanisms of diapause. To better understand the biology and diapause mechanism of the insect pest species, *D. antiqua*, the transcriptome was sequenced using Illumina paired-end sequencing technology. Approximately 54 million reads were obtained, trimmed, and assembled into 29,659 unigenes, with an average length of 607 bp and an N50 of 818 bp. Among these unigenes, 21,605 (72.8%) were annotated in the public databases. All unigenes were then compared against *Drosophila melanogaster* and *Anopheles gambiae*. Codon usage bias was analyzed and 332 simple sequence repeats (SSRs) were detected in this organism. These data represent the most comprehensive transcriptomic resource currently available for *D. antiqua* and will facilitate the study of genetics, genomics, diapause, and further pest control of *D. antiqua*.

The onion maggot *Delia antiqua* is a major insect pest of cultivated vegetables, especially onions, and is widely distributed in the northern hemisphere. It can be induced into summer and winter diapauses, both happening at the pupal stage and just after head evagination (Ishikawa *et al.* 2000). These characteristics make it a good model for investigating the molecular mechanisms of pupal diapause (Hao *et al.* 2012). To date, there have been no efforts to sequence the complete transcriptome of *D. antiqua*. The research on this species is limited by a very small amount of genomic data. As of February 2014, only 245 expressed sequence tags (ESTs) have been deposited in GenBank. To better understand the biological basis of diapause and *D. antiqua*, there is a need to explore genomic biology in *D. antiqua*.

Advances in next-generation sequencing (NGS) and assembly algorithms have rapidly promoted the development of next-generation RNA sequencing (RNA-seq). RNA-seq can reveal the entire transcriptome in a selected tissue and species of interest and generates quantitative expression scores for each transcript (Wilhelm and Landry 2009). Such transcriptome analysis will likely replace large-scale microarray approaches (Marioni *et al.* 2008; Simon *et al.* 2009) because of its lower cost and greater sequence yield. This allows the measurement of transcriptome composition and quantitatively surveys RNA expression patterns (Wilhelm and Landry 2009). It also addresses comparative genomic-level questions and allows the development of molecular markers (Parra-Gonzalez *et al.* 2012). In the past several years, RNA-seq has been used on many genomes, from bacteria, archaea, and lower eukaryotes to higher eukaryotes. It is particularly effective when a reference genome is not available (Martin and Wang 2011).

The main objective of our study was to construct a reference transcriptome of *D. antiqua* for future genetic and genomic studies of this species. Here, we present a comprehensive analysis of the *de novo* transcriptome sequencing results for cDNA samples derived from eggs, larvae, pupae, and adults of *D. antiqua* (covering every stage of development) by using the Illumina Hiseq2000 sequencing platform. As a result, a total of 29,659 unigenes were assembled and identified; among them, 21,050 were annotated. Based on the transcriptome, codon usage bias was analyzed and simple sequence repeats (SSRs) were detected. To our knowledge, this is the first report of the complete transcriptome and transcriptome characteristics of *D. antiqua*. This new dataset will provide a useful resource for future studies of the genetics and genomics of this species.

## Materials and Methods

### Insect rearing and RNA extraction

The non-diapausing colony (ND) of *D. antiqua* was maintained in the Institute of Entomology and Molecular Biology, Chongqing Normal University, China at 20 ± 0.2° under 50–70% relative humidity (RH) with a 16L:8D photocycle as previously described (Chen *et al.* 2005). Larvae were raised at 25 ± 0.5° under 50–70% RH with 16L:8D to induce summer diapause (SD). Newly formed puparia were used for experiments. They were kept under the same conditions as the larvae until day 15 after pupariation and then were transferred to 16° and 16L:8D to trigger diapause termination. For winter diapause (WD) induction, the conditions were 15 ± 0.5° under 50–70% RH with a 12L:12D through larvae and pupae. Ten samples were taken and then immediately frozen in liquid nitrogen for later RNA extraction. These 10 samples include ND eggs (from every incubation day), ND larvae (from every larval instar), ND pupae (female and male, from the stage equal to diapause development), ND adults (female and male, from different developmental stages), three SD pupae samples (female and male, sampled separately at diapause initiation, maintenance, and termination), and three WD pupae samples (female and male, sampled separately at diapause initiation, maintenance, and termination) of *D. antiqua*.

Total RNA was separately extracted from the 10 samples using TRIzol Reagent (Invitrogen, Carlsbad, CA) following the manufacturer’s protocol. To eliminate genomic DNA, the RNA samples were treated with RNase-Free DNase I according to the manufacturer’s protocol (Qiagen, USA). The RNA integrity was confirmed using the Agilent 2100 Bioanalyzer with a minimum integrity number value of 7; 13% of total RNA each from ND eggs, ND larvae, and ND adult samples and 8.7% of total RNA from each of the other seven samples were pooled together for cDNA preparation.

### mRNA purification and cDNA synthesis and Illumina sequencing preparation and sequencing

Beads with Oligo(dT) were used to isolate poly(A) mRNA after total RNA extraction. Fragmentation buffer was added for cutting mRNA into short fragments. Taking these short fragments as templates, random hexamer-primer was used to synthesize the first-strand cDNA. The second-strand cDNA was synthesized using buffer, dNTPs, RNaseH, and DNA polymerase I. Short fragments were purified with QiaQuick PCR extraction kit (Qiagen, USA) and resolved with EB buffer for end reparation and tailing A. After that, the short fragments were connected with sequencing adapters. After agarose gel electrophoresis, the ligated products were purified and amplified with PCR to create the final cDNA library. The cDNA library was sequenced by Beijing Genomics Institute (BGI)-Shenzhen, Shenzhen, China, using Illumina HiSeq 2000, according to the manufacturer’s instructions.

### *De novo* transcriptome assembly

The raw reads produced from sequencing machines were cleaned by removing adapter sequences, ambiguous reads (reads with unknown nucleotides “N” larger than 5%), and low-quality sequences (reads with more than 10% Q<20 bases). The quality reads were assembled into unigenes using the short reads assembling program Trinity (Grabherr *et al.* 2011). Reads with a certain length of overlap area were first joined to form longer fragments, which are called contigs without gaps. Then, the reads were mapped back to contigs; with paired-end reads it is possible to detect contigs from the same transcript, as well as the distances between these contigs. Next, Trinity connects the contigs and obtains sequences that can no longer be extended. Such sequences are defined as unigenes. Assembled sequences less than 200 nt were deleted. Finally, unigenes were divided into two classes by gene family clustering. One class includes clusters in which several unigenes with a similarity higher than 70% are classified into one cluster with the prefix CL. The other class includes singletons with the prefix Unigene. FPKM for samples was calculated to show expression quantity, thus avoiding the influence of sequencing length and difference (Mortazavi *et al.* 2008). Each FPKM was log_10_ transformed.

### Functional annotation

The generated unigenes larger than 200 nt were searched against NCBI non-redundant protein database (Nr), Swiss-Prot, Kyoto Encyclopedia of Genes and Genomes (KEGG), Cluster of Orthologous Groups (COG), and Gene Ontology (GO) databases using BLASTX alignment (E-value ≤1e−5), and against NCBI non-redundant nucleotide database (Nt) by BLASTN (E-value ≤1e−5). The best-aligned results were used to decide sequence direction and the coding sequence (CDS) of unigenes. If results of different databases conflicted with each other, then *a priori*ty order of Nr, Nt, Swiss-Prot, KEGG, and COG was followed when deciding sequence direction of unigenes. ESTScan (Iseli *et al.* 1999) was used to predict the sequence direction and CDS when unigenes were unaligned to any of the databases. GO annotations of the unigenes were determined by the Blast2GO program (Conesa *et al.* 2005). After obtaining GO annotation for each unigene, WEGO software (Ye *et al.* 2006) was used to display GO functional classification.

### Characterization of open reading frames and codon usage

The open reading frames (ORFs) in each unigene sequence were predicted by searching against protein databases using BLASTX (E-value ≤1e−5) in the following order: Nr, SwissProt, KEGG, and COG. Sequences having hits in the former database did not go to the next round of searching against a later database. The coding regions were then extracted according to the best BLASTX match with a custom Perl script. CodonW (http://codonw.sourceforge.net/) (Peden 1999) was used to analyze RSCU and GC3 of ORFs (≥150 nt). Perl scripts were used to process the output files of CodonW. We used SPSS 13.0 statistics software (SPSS Inc., Chicago, IL) for correlation analysis.

NC and CAI were calculated by DAMBE (Xia 2013) using the *Delia antiqua* ribosomal proteins codon usage table as the reference when calculated CAI.

### SSR detection

SSRs Identification Tool (SSRIT; http://www.gramene.org/db/markers/ssrtool) (Temnykh *et al.* 2001) was used for detection of SSRs on unigenes of *D. antiqua* longer than 1 kb. The parameters were designed for identifying perfect di-, tri-, tetra-, penta-, and hexa-nucleotide motifs, with minimum thresholds of six, five, four, four, and four repeats, respectively. Mononucleotide repeats were not considered because of the possibility of the Illumina homopolymer sequencing problem associated with this technology.

### Accession code

The clean reads produced in this study have been deposited at DDBJ/EMBL/GenBank Short Read Archive under project number PRJNA208983, BioSample number SAMN02208942, and accession code SRR916227. This Transcriptome Shotgun Assembly project has been deposited at DDBJ/EMBL/GenBank under the accession GAWI00000000. The version described in this article is the first version, GAWI01000000.

## Results and Discussion

### Illumina sequencing and assembly

To achieve an overall understanding of the *D. antiqua* transcriptome, a mixed cDNA sample obtained from diverse developmental stages of this species was prepared and sequenced using the Illumina Hiseq2000 sequencing platform. Each sequenced sample yielded 2×50-nt independent reads from either end of a cDNA fragment. We obtained a total of 53.81 million raw reads. After the removal of raw reads that only had adaptor fragments and ambiguous and low-quality reads, 51.50 million (4.63 Gnt, 95.7% of the raw reads) clean reads remained, with a Q20% of 98.2%, GC content of 39.03%, and unknown nucleotide “N” of 0.00%. An overview of the sequencing, assembling, and functional annotation results is presented in Table 1.

**Table 1:** Statistics from RNA-seq–based sequencing, assembling, and functional annotation for *D. antiqua*

Sequencing results	N of total raw reads	53,814,782
	N of total clean reads	51,497,228
	N of total clean nucleotides (nt)	4,634,750,520
	Q20 percentage of total clean reads	98.24%
	GC percentage of total clean nucleotides	39.03%
	N percentage of total clean nucleotides	0.00%
**Assembling results**	N of unigenes	29,659 (4507 into distinct clusters; 25,152 singletons)
	Total length (nt) of total unigenes	18,008,540
	Mean length (nt) of total unigenes	607
	N50 (nt) of total unigenes	818
**Annotation**	Unigenes with Nr database	21,050 (71.0%)
(E-value≤1e−5)	Unigenes with Nt database	10,058 (33.9%)
	Unigenes with Swiss-Prot database	15,886 (53.6%)
	Unigenes with KEGG database	14,147 (47.7%), 257 pathways
	Unigenes with COG database	6600 (22.3%), 25 functional categories
	Unigenes with GO database	13,238 (44.6%), 60 subcategories belonging to 3 main categories
	Biological process	25 subcategories
	Cellular component	18 subcategories
	Molecular function	17 subcategories
	Total unigenes annotated	21,605 (72.8% of 29,659 unigenes)

These clean data were assembled *de novo* by Trinity, producing 29,659 unigenes longer than 200 nt (18 Mnt), with an average length of 607 nt and an N50 of 818 nt (Table 1). Of these, 4507 (15.2%) could be classified into distinct clusters, 25,152 (84.8%) were distinct singletons, 11,705 unigenes (39.5%) were longer than 500 nt, and 5530 unigenes (18.6%) were longer than 1000 nt. The length distributions of unigenes are shown in Supporting Information, File S1. To test the assembly quality, 276 *D. antiqua* available cDNA (245 ESTs downloaded and 31 cDNA cloned in our laboratory) sequences were used as queries to BLAST all unigenes generated in our study with a stringent E-value of 1e−7. 227 queries having hits, giving 82.2% (227/276) coverage.

### Functional annotation

BLASTX alignments were conducted between the predicted protein sequences and several public databases, including Nr, NCBI Nt, Swiss-Prot, GO database, COG database, and the Kyoto Encyclopedia of Genes, and Genomes (KEGG) database, with an E-value threshold of 1e−5. The results indicate that out of 29,659 unigenes, a total of 21.050 (71.0%) unigenes were annotated against Nr, 10,058 (33.9%) were annotated against Nt, 15,886 (53.6%) were annotated against Swiss-Prot, 13,238 (44.6%) were annotated against GO database, 6600 (19%) were annotated against COG database, and 14,147 (43%) were annotated against the KEGG database obtained annotations (Table 1). Altogether, BLAST searches against Nr, Nt, Swiss-Prot, GO, COG, and KEGG databases showed that a total of 21,605 (72.8% of 29,659) identified unigenes could be annotated with known biological functions.

**Figure 1:**
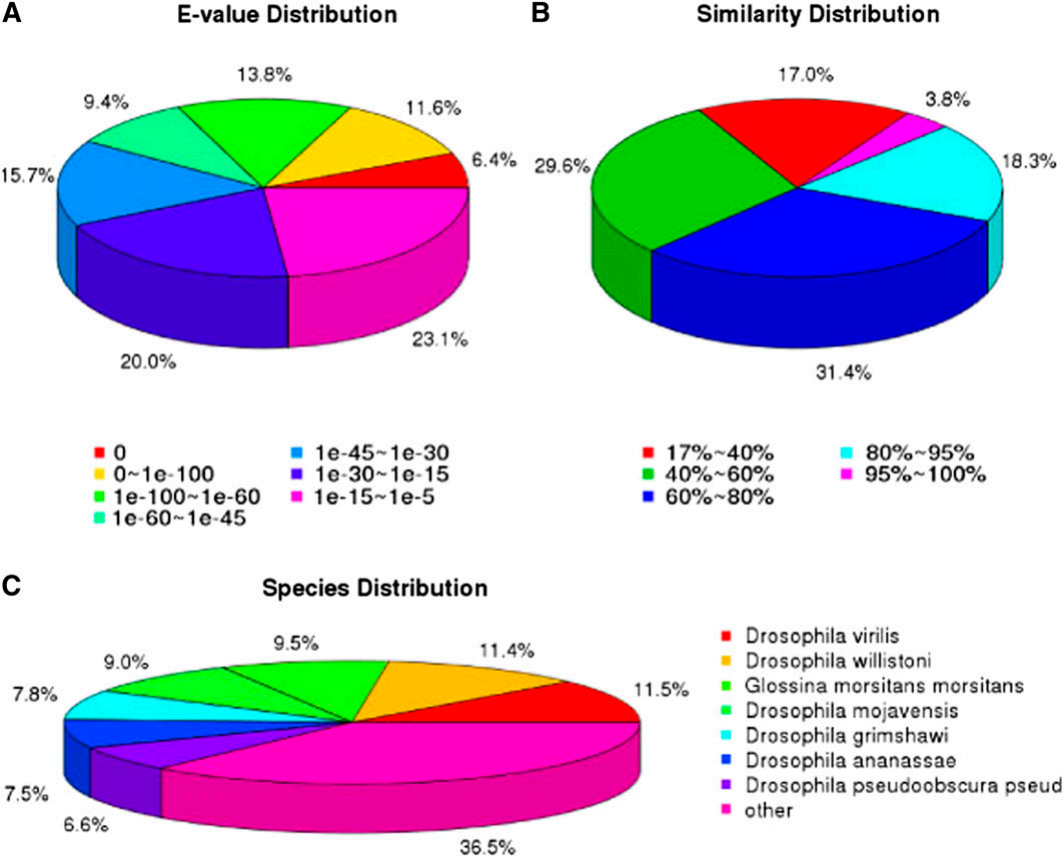
NR classification of *D. antiqua* unigenes. (A) E-value distribution. (B) Similarity distribution. (C) Species distribution.

After Nr database annotation, the E-value distribution, identity distribution, and species distribution were then analyzed (Shi *et al.* 2011) (Figure 1). For the E-value distribution of the predicted proteins, the top hits indicated that 41.2% of the mapped sequences had a significant similarity with a stringent threshold of less than 1e−45, and 58.8% of the similar sequences ranged from 1e−5 to 1e−45 (Figure 1A). For the similarity distribution of the predicted proteins, most of the sequences (53.5%) had a similarity higher than 60%, and 22.1% of the sequences had a similarity higher than 80% (Figure 1B). The species distribution showed that *D. antiqua* genes had the greatest number of matches with *Drosophila* genes. Among them, 11.5% unigenes had best matches and first hit against *Dr. virilis* sequences, 11.2% against *Dr. willistoni* and 9.5% against *Glossina morsitans*, followed by other insect species (9.0% *Dr. mojavensis*, 7.8% *Dr. grimshawi*, 7.5% *Dr. ananassae*, 6.6% *Dr. pseudoobscura*, and 36.5% other species) (Figure 1C).

**Figure 2:**
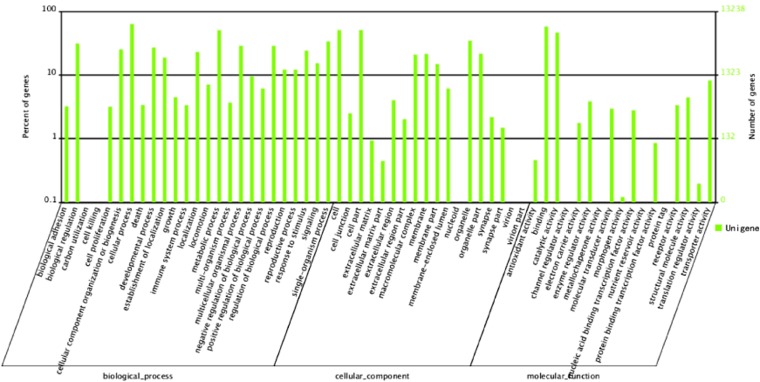
GO function classification of *D. antiqua* unigenes.

GO terms were assigned to *D. antiqua* unigenes for functional categorization; 13,238 unigenes were categorized into 60 subcategories belonging to 3 main categories, including biological process (25), molecular function (17), and cellular component (18) after assignment (Table 1). In the category of biological processes, the dominant GO terms were grouped into cellular processes and metabolic processes. Among the 18 subcategories of cellular components, those assignments were mostly given to cell part and organelle. Within the molecular function category, there was a high percentage of genes with binding, catalytic activity, and transporter activity (Figure 2). These GO annotations revealed that diverse structural, regulatory, metabolic, and transporter proteins were encoded by expressed genes in *D. antiqua*.

**Figure 3:**
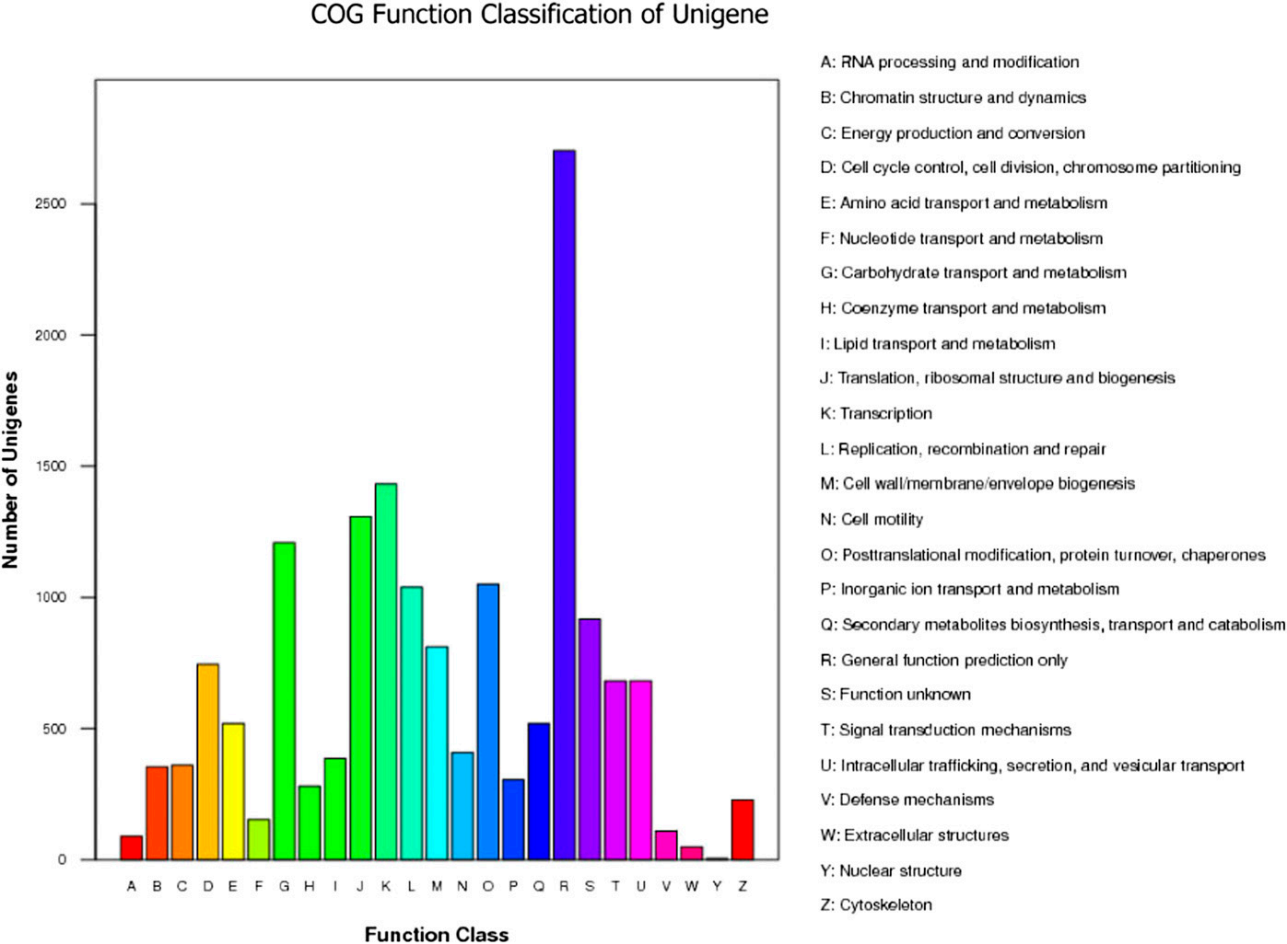
COG classification of *D. antiqua* unigenes.

COG is a database that classifies gene products into different clusters of orthologous groups. As a result of a search of 29,659 unigenes against the COG database for orthologous genes, 6600 *D. antiqua* unigenes were classified into 25 functional categories. The four largest categories were the following: general function prediction only (2703; 40.1%); transcription (1431; 21.7%); translation, ribosomal structure, and biogenesis (1308; 19.8%); and carbohydrate transport and metabolism (1209; 18.3%). The smallest groups were defense mechanisms (109; 1.7%), RNA processing and modification (88; 1.3%), extracellular structures (48; 0.7%), and nuclear structure (3; 0.04%) (Figure 3).

Based on the KEGG database, the potential involvement of the assembled unigenes in biological pathways was annotated with corresponding Enzyme Commission (EC) numbers and pathways, providing intracellular metabolic pathways and functions of gene products of unigenes. After searching 29,659 assembled unigenes against KEGG, a total of 14,147 unigenes were assigned to 257 KEGG pathways (Table 1). The pathways most represented by unique sequences were metabolic pathways with 1935 unigenes (13.7%) that were associated with basic metabolic functions, followed by focal adhesion (503; 3.6%), pathways in cancer (501; 3.5%), RNA transport (445; 3.1%), and regulation of actin cytoskeleton (409; 2.9%). These functional annotations provide a basis for exploring specific biological processes, functions, subcellular localization, and pathways of gene products in *D. antiqua* research.

### Comparative analysis with *D. antiqua* and other Dipteran genomes

**Figure 4:**
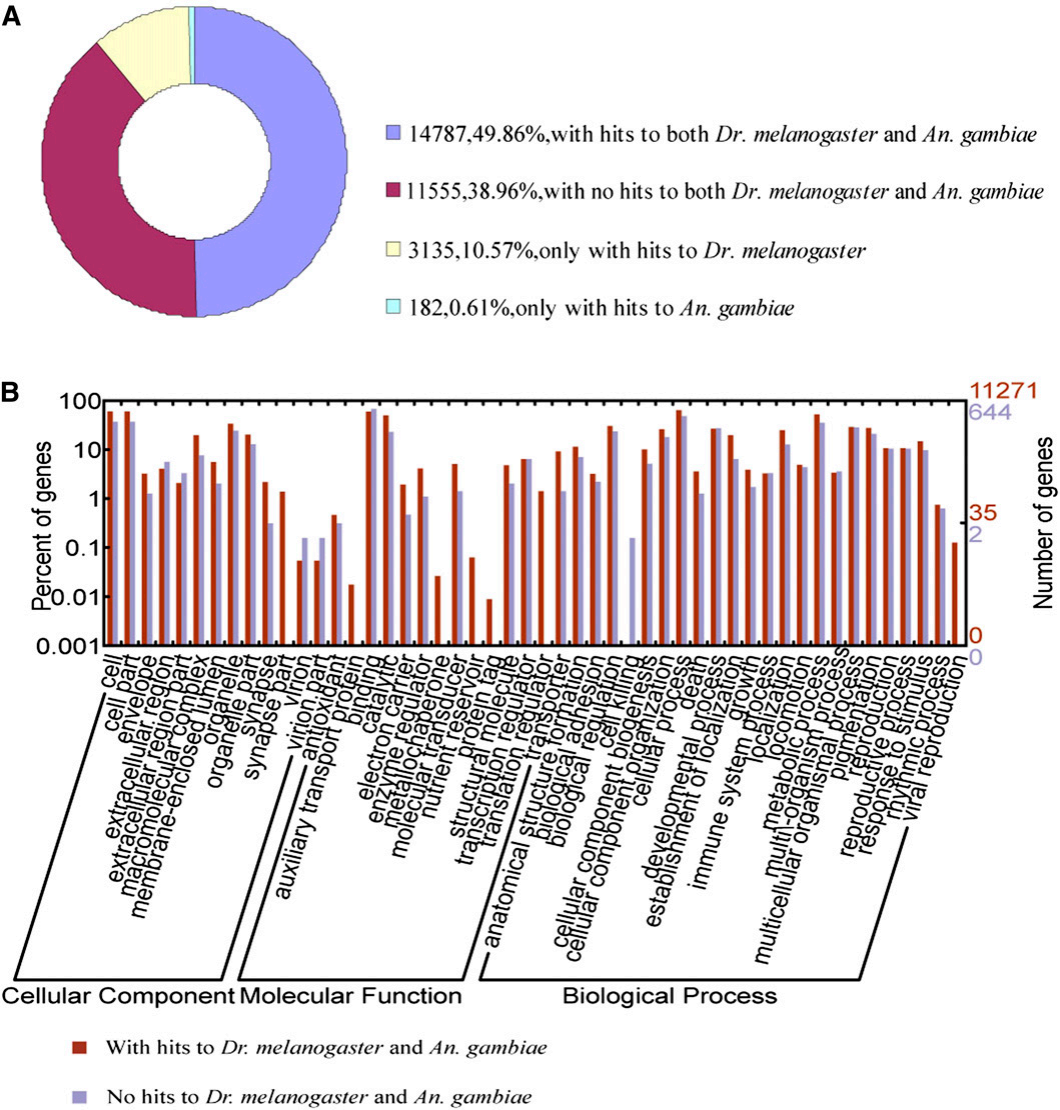
* D. antiqua* unigenes similarity comparison with *Dr. melanogaster* and *An. gambiae* and functional classification by GO analysis. (A) Similarity search of *D. antiqua* sequences against *Dr. melanogaster* and *An. Gambiae*. (B) Functional classification of *D. antiqua* unigenes with and without homologs with *Dr. melanogaster* and *An. gambiae*.

To further understand the relationship between *D. antiqua* and two other Dipteran model species, *Dr. melanogaster* and *An. gambiae*, and to identify unigenes that might be unique to *D. antiqua*, BLASTX was used for the comparisons of *D. antiqua* to *Dr. melanogaster* and *An. gambiae* with a cut-off E-value ≤1e−5, because good BLAST hits are more easily obtained with amino acid sequences than with nucleotide sequences. Comparisons of unigenes to amino acid sequences of *Dr. melanogaster* and *An. gambiae* (E-value <1e−5 in BLASTX) showed that 17,922 (60.4%) *D. antiqua* unigenes had similarity hits to *Dr. melanogaster* peptides and 14,969 (50.5%) unigenes had similarity hits to *An. gambiae* peptides. Among these aligned sequences, 14,787 unigenes had similarities with both *Dr. melanogaster* and *An. gambiae*, whereas 3135 (10.57%) unigenes only had similarity hits to *Dr. melanogaster* and 182 (0.61%) unigenes only had similarity hits to *An. gambiae* (Table 2 and Figure 4A). A total of 11,555 (38.96%) *D. antiqua* unigenes did not match any *Dr. melanogaster* and *An. gambiae* sequences and were assumed to be unique to *D. antiqua* (Table 2 and Figure 4A).

**Table 2:** Comparative analysis between *D. antiqua* and other Dipteran genomes using BLASTX with a cut-off E-value of 1E−5

	*Drosophila melanogaster*	*Anopheles gambiae*
N of a.a. sequences	27,538	14,324
N of sequences with a.a. > 50	27,410	14,296
Genome sequence version	r5.47	AgamP3.6
Source of genome sequence	Flybase	Vectorbase
With hits to	17,922 (60.43%)	14,969 (50.47%)
Only with hits to	3135 (10.57%)	182 (0.61%)
With hits to both	14,787 (49.86%)	
With no hits to both	11,555 (38.96%)	

After the similarity search, GO functional classification was used to compare two groups of *D. antiqua* unigenes, shared homologs with *Dr. melanogaster* and *An. gambiae* and unigenes unique to *D. antiqua* (Figure 4B). In all, among 14,787 shared homologs, there were 11,271 unigenes that were assigned one or more GO terms. The GO analysis showed that for biological processes, genes involved in cellular processes and metabolic processes were highly represented. For molecular functions, binding activity was the most represented GO term, followed by catalytic activity. Regarding cellular components, the most represented categories were “cell” and “cell part.” Only 644 of 11,555 unigenes predicted to be unique to *D. antiqua* were annotated by GO analysis. This low annotation percent is probably attributable to the relatively small fraction of *D. antiqua* gene information available in public databases, especially compared with *Dr. melanogaster* and *An. gambiae*.

There are some differences between the annotations of shared homologs and *D. antiqua* unique unigenes, such as the GO term “synapse part” in the cellular component, “auxiliary transport protein,” “metallochaperone,” “nutrient reservoir,” and “protein tag” in molecular functions, and “cell killing” and “viral reproduction” in biological process annotation. We postulate that differences in function allocation of the *D. antiqua* unigenes with sequence similarity hits to *Dr. melanogaster* and *An. gambiae* contribute to the divergence of *D. antiqua* from other insects. However, *D. antiqua* is famous for its diapause. The large bulk of predicted unigenes unique to *D. antiqua* represent a valuable resource to explore *D. antiqua* gene diversity and provide a basis for finding the genes involved in the specific physiological processes of diapause.

### Codon usage bias

Codon usage bias is a phenomenon in which specific codons have unequal usage frequencies compared to other synonymous codons during the translation of genes. It provides a useful tool in functional genomic research and helps illuminate the physiological, biochemical, and molecular mechanism of a gene or genome (Angov 2011). Factors that influence the extent of codon usage bias include expression level, GC content, codon position, gene length, environmental stress, and population size (Behura and Severson 2012).

In our study, a total of 20,578 predicted ORFs longer than 150 nt in the *D. antiqua* transcriptome were used in the codon usage bias analyses. Total counts of codons and the relative synonymous codon usage (RSCU) for these sequences were calculated. Average RSCU values showed that the seven most frequently used codons in *D. antiqua* are CGU, UUA, GGU, UUG, GAU, AAU, and GCU. The seven seldom used codons in *D. antiqua* are GGG, CGG, CUC, GCG, AGG, GAC, and AAC, without considering stop codons (File S2). The results are very similar to the results obtained from Dipteran insects based on the genome-specific frequencies of the codons (Behura and Severson 2011).

The average GC content of 20,578 ORFs was 39.14% and average GC content of all 29,659 unigenes in *D. antiqua* was 39.03%. These values are different from the GC content in the *An. gambiae* (55.8%) and *Dr. melanogaster* (53.9%) genomes (Zagrobelny *et al.* 2009). Different species possess different GC content at the third codon position (GC3), which largely decides genome base composition. Our results showed the average GC3 was 28.36% in *D. antiqua* (File S3).

The effective number of codons (Nc) quantifies how far the codon usage of a gene departs from equal usage of synonymous codons (Fuglsang 2004) and is a measure of codon usage biases in genes and genomes that ranges from 20 (maximal bias) to 61 (unbiased) (Wright 1990; Sun *et al.* 2013). *D. antiqua* shows a medium degree of codon usage bias, as measured by the mean Nc value (45.06) (File S3). An Nc plot (plot of Nc *vs.* GC3) shows the relationship between codon usage bias and GC3 and is widely used to study the codon usage variation among genes in different genomes (Fuglsang 2008). In an Nc plot, if the values of Nc fall on the continuous curve between Nc and GC3, then the codon usage variation among the genes was only determined by variation in GC3 content. In our study, most of the genes have an Nc value lower than expected on the curve, fall within a restricted cloud at GC3 between 0.056 and 0.928, and have Nc values between 33.97 and 56.21 (File S4). The Nc plot shown here implies that the codon usage of a large number of genes in *D. antiqua* is subject to other factors.

Gene expression level can heavily influence codon usage (Behura and Severson 2012). For instance, genes encoding ribosomal proteins are known to be highly expressed and tend to have more biased usage than those with low expression (Hershberg and Petrov 2008). Significant positive correlation between gene expression and codon usage bias has been studied in microarray data from many species (Behura and Severson 2011). To test the correlation between gene expression and codon bias, Codon Adaptation Index (CAI) (Xia 2007) values and gene expression levels (log_10_ FPKM) for each ORF (total of 20,578 ORFs) were compared. A significantly positive correlation between the CAI and gene expression levels was observed (Pearson correlation: r = 0.126; *P* =1.28e−73) (File S3 and File S5).

In this study, we calculated total codon usages and identified the most frequent and most seldom used codons. The influences of GC3 and expression level on codon usage bias in *D. antiqua* were also tested. A large number of genes have an Nc value lower than the expected value located on the curve in the Nc plot, implying that the codon usage of many *D. antiqua* genes are not just determined by GC3. A significant correlation between expression levels and codon usage was observed in *D. antiqua*. Knowledge of the codon usage pattern obtained in *D. antiqua* could help us understand mechanisms of codon usage bias and improve exogenous gene expression in future transgenic manipulation, and thus serves as a useful tool in functional genomic research in *D. antiqua*.

### SSR discovery

In the *D. antiqua* transcriptome, 4637 unigene sequences longer than 1 kb were used for SSR identification; 315 sequences containing a total of 352 SSRs of 94 kinds were identified, with 37 of the sequences containing more than 1 SSR (Table 3). The frequency of SSR in the *D. antiqua* transcriptome was 1 per 14.7 kb. The most abundant repeat motif was the tri-nucleotide, accounting for 82.7%, followed by the di-nucleotide repeat motif (14.7%) and tetra-nucleotide (2.5%) repeat units (Table 4). We also calculated the frequencies of SSRs with different numbers of tandem repeats (Table 4). SSRs with five tandem repeats (69.9%) were the most common, followed by six tandem repeats (21.6%), seven tandem repeats (5.4%), eight tandem repeats (1.1%), and four tandem repeats (1.7%). A detailed list of SSRs identified is shown in File S6.

**Table 3:** Features of SSRs identified in the *D. antiqua* transcriptome

Total n of examined unigenes	29,659
N of unigenes longer than 1 kb	4637
Total nucleotides screened (knt)	7423
N of unigenes containing SSRs	315
N of identified SSRs	352
Kinds of identified SSRs	94
N of unigenes containing more than 1 SSRs	37
Frequency of SSR in transcriptome	1/14.7 kb

**Table 4:** Frequency of SSRs in *D. antiqua* transcriptome

N of Nucleotides	N of Motif Repeats									
	4	5	6	7	8	9	10	>10	Total	%
Di	—	—	40	10	1	1	—	—	52	14.7
Tri	—	245	34	9	3	—	—	—	291	82.7
Tetra	6	1	2	—	—	—	—	—	9	2.5
Penta	—	—	—	—	—	—	—	—	0	0
Hexa	—	—	—	—	—	—	—	—	0	0
Total	6	246	76	19	4	1	0	0	352	
%	1.7	69.9	21.6	5.4	1.1	0.3	0	0		

SSR markers are useful tools for assessing genetic variation and relationships in genetic mapping studies (Luikart *et al.* 2003). SSR markers developed from transcriptome data are cheaper when compared with traditional isolation of genomic DNA-derived SSRs, because large-scale transcriptome sequencing programs based on NGS methods produce large amounts of sequence data. Moreover, because transcriptome-based SSRs mainly occur in the protein-coding regions of annotated unigenes, they are better for identifying associations with functional genes and thus with phenotypes (Zalapa *et al.* 2012).

Efficient identification of transcriptome-based SSRs has been reported for many organisms, such as *Ma Bamboo* (Liu *et al.* 2012) and *Spodoptera exigua* (Pascual *et al.* 2012), but no studies have been reported for *D. antiqua*. The unigenes obtained from *D. antiqua* have provided a good resource for SSR mining. Different software (such as SSRIT and SSRPrimer) (Temnykh *et al.* 2001; Robinson *et al.* 2004), and parameters used in detection may produce minor influences on the efficiency of detection. In our study, 352 SSRs were identified and the tri-nucleotide repeat motif was the most abundant form of SSR repeat, consistent with the results from another insect species, *Sp. exigua* (Pascual *et al.* 2012). The SSR markers identified from *D. antiqua* transcriptome will serve as potential markers for genome mapping and identification of important functional genes in this species.

## Conclusions

The onion maggot *D. antiqua* is a major insect pest of cultivated vegetables and a good model for summer and winter diapause studies. However, study of this species was restricted by the availability of research data and scientific resources. To establish a genomic resource, we used the Illumina Hiseq2000 sequencing platform to sequence the *D. antiqua* transcriptome and produced 29,659 assembled unigenes, of which 21,050 (71%) were annotated. This study dramatically increased the number of genes from *D. antiqua* and will facilitate future genomic-level studies in this species. To our knowledge, our results represent approximately 120-fold more genes than all *D. antiqua* genes deposited in GenBank (as of December 2012). Some showed homology with other Dipteran genomes, which is of significance for evolutionary studies of Diptera. Other members that did not match *Dr. melanogaster* or *An. gambiae* sequences were assumed to be unique to *D. antiqua*, offering a valuable resource for gene diversity research in *D. antiqua*. Analysis of codon usage bias in *D. antiqua* may be helpful for understanding the mechanisms of codon usage and for exogenous gene expression in this species. Furthermore, the transcriptome-based SSR markers identified in *D. antiqua* will help identification of pest-related genes and contribute to genome mapping and pest control. We believe that results obtained from this study will act as a useful genomic resource to accelerate explorations of the molecular mechanisms of pest adaptation, diapause, and functional genomics in this important species.

## Supplementary Material

Supporting Information
