# Supplementary material for: The de novo Transcriptome and Its Analysis in the Worldwide Vegetable Pest, Delia antiqua (Diptera: Anthomyiidae)
Source: G3 (Bethesda). 2014 Mar 10;4(5):851–9. doi: 10.1534/g3.113.009779 (PMC4025484; doi:10.1534/g3.113.009779)
Supplement: Supporting Information [file supp_4_5_851__index.html]

The De Novo Transcriptome and Its Analysis in the Worldwide Vegetable Pest, Delia antiqua (Diptera: Anthomyiidae) — The de novo Transcriptome and Its Analysis in the Worldwide Vegetable Pest, Delia antiqua (Diptera: Anthomyiidae) — Supporting Information 

# The *de novo* Transcriptome and Its Analysis in the Worldwide Vegetable Pest, *Delia antiqua* (Diptera: Anthomyiidae)

## Supporting Information for Zhang *et al.*, 2014

**Files in this Data Supplement:**

- Supporting Information - Files S1-S6 (PDF, 240 KB)
- File S1 - Length distribution of unigenes in *D. antiqua*. (PDF, 144 KB)
- File S2 - Total codon usage and codon usage bias in *D. antiqua* transcriptome. (PDF, 144 KB)
- File S4 - A plot of Nc versus GC3 (Nc-plot) for *D. antiqua* ORFs. (PDF, 123 KB)
- File S5 - Relationship between CAI (Codon Adaptation Index) and expression level (Log10 (FPKM)) of all *D. antiqua* transcriptome unigenes. (PDF, 147 KB)
- File S3 - The ID and FPKM of unigenes, and the CAI, Nc and GC3 of ORFs in each unigene in *D. antiqua*. (.xls, 3 MB)
- File S6 - SSR identification of *D. antiqua* unigenes. (.xls, 58 KB)
