# Supplementary material for: The de novo Transcriptome and Its Analysis in the Worldwide Vegetable Pest, Delia antiqua (Diptera: Anthomyiidae)
Source: G3 (Bethesda). 2014 Mar 10;4(5):851–9. doi: 10.1534/g3.113.009779 (PMC4025484; doi:10.1534/g3.113.009779)
Supplement: Supporting Information [file supp_g3.113.009779_009779SI.pdf]

G3: Genes | Genomes | Genetics

The *de novo* transcriptome and its analysis of the worldwide vegetable pest, *Delia antiqua* (Diptera: Anthomyiidae)

Yujuan Zhang, Youjin Hao, Fengling Si, Shuang Ren, Ganyu Hu, Li Shen, Bin Chen*

Institute of Entomology and Molecular Biology, College of Life Sciences, Chongqing Normal University, Chongqing, P.R. China.

*Correspondence E-mail: c_bin@hotmail.com

DOI: 10.1534/g3.113.009779

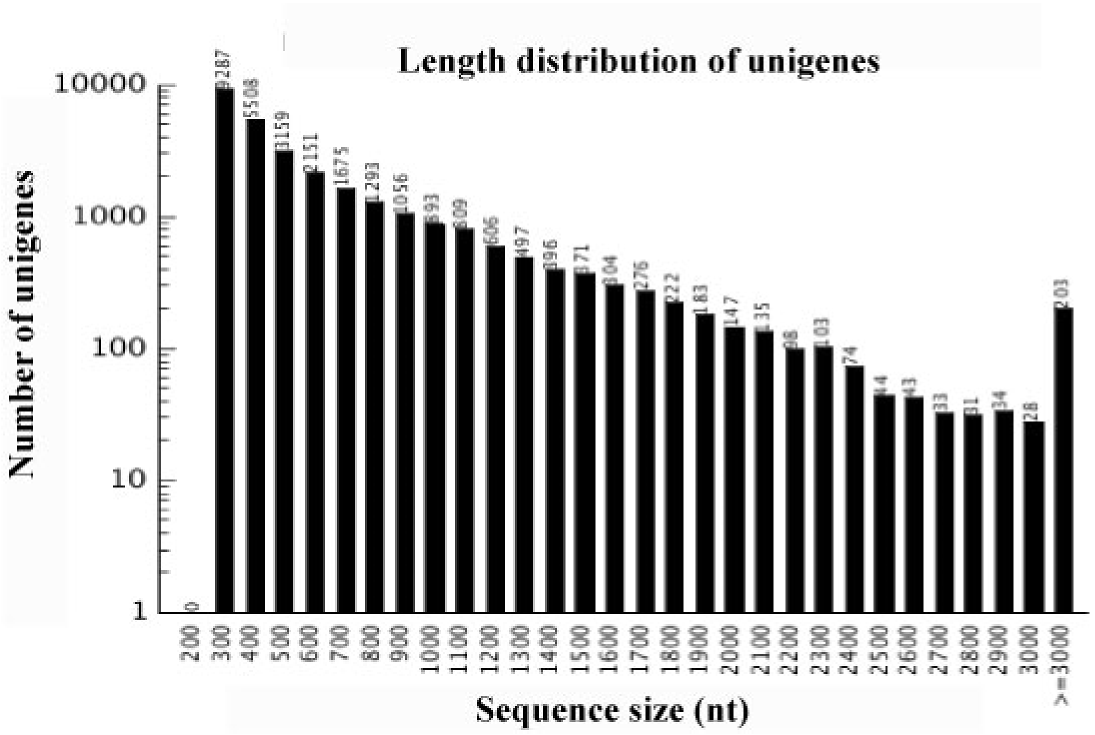

File S1 Length distribution of unigenes in *D. antiqua*.

File S2 Total codon usage and codon usage bias in *De. antiqua* transcriptome. A total of 20578 ORF containing 3913673 codons in the *De. antiqua* transcriptome were used in the calculation of codon usage bias measured by RSCU.

Codon	Amino Acid	Total codon counts	RSCU	Codon	Amino Acid	Total codon counts	RSCU
**CGU**	**R**	81490	**2.037**	**UCC**	**S**	43072	0.737
**UUA**	**L**	118878	**1.808**	**GGA**	**G**	44472	0.733
**GGU**	**G**	111980	**1.623**	**CGC**	**R**	27367	0.686
**UUG**	**L**	104910	**1.477**	**GUG**	**V**	43091	0.657
**GAU**	**D**	172863	**1.342**	**UCG**	**S**	39066	0.644
**AAU**	**N**	177112	**1.333**	**CUA**	**L**	37341	0.569
**GCU**	**A**	105216	**1.299**	**UGG**	**W**	38282	0.549
**GAA**	**E**	202421	1.286	**CUU**	**L**	34950	0.532
**CAA**	**Q**	147888	1.271	**CGA**	**R**	18777	0.525
**GUU**	**V**	83193	1.247	**GUC**	**V**	30508	0.445
**AAA**	**K**	195467	1.234	**UUC**	**F**	43668	0.435
**UAU**	**Y**	99910	1.223	**AGC**	**S**	25606	0.430
**ACA**	**T**	87752	1.199	**AAG**	**K**	72085	0.417
**CCA**	**P**	73462	1.188	**CUG**	**L**	26604	0.393
**CAU**	**H**	75842	1.159	**CCG**	**P**	22061	0.384
**UUU**	**F**	107042	1.159	**UGC**	**C**	21035	0.382
**UCA**	**S**	69422	1.143	**ACG**	**T**	25727	0.368
**AGA**	**R**	41095	1.142	**CAG**	**Q**	44046	0.363
**AGU**	**S**	64397	1.112	**CAC**	**H**	23232	0.358
**AUU**	**I**	104368	1.112	**GAG**	**E**	55560	0.351
**AUA**	**I**	89316	1.024	**UAC**	**Y**	29080	0.348
**ACU**	**T**	73298	1.014	**AUC**	**I**	31938	0.348
**UCU**	**S**	59447	0.971	**AAC**	**N**	46335	**0.328**
**GCC**	**A**	75178	0.970	**GAC**	**D**	37400	**0.287**
**GUA**	**V**	62064	0.961	**AGG**	**R**	9837	**0.282**
**UGU**	**C**	52258	0.950	**GCG**	**A**	18599	**0.267**
**CCC**	**P**	53246	0.912	**CUC**	**L**	16359	**0.244**
**GCA**	**A**	57320	0.798	**CGG**	**R**	7571	**0.206**
**CCU**	**P**	48604	0.795	**GGG**	**G**	8038	**0.140**
**GGC**	**G**	51205	0.778	**UAA**	*****	685	0.029
**ACC**	**T**	54346	0.753	**UGA**	*****	679	0.023
**AUG**	**M**	89348	0.744	**UAG**	*****	264	0.011

File S3

The ID and FPKM of unigenes, and the CAI, Nc and GC3 of ORFs in each unigene in *D. antiqua*.

File S3 is available for download as an Excel file at http://www.g3journal.org/lookup/suppl/doi:10.1534/g3.113.009779/-/DC1

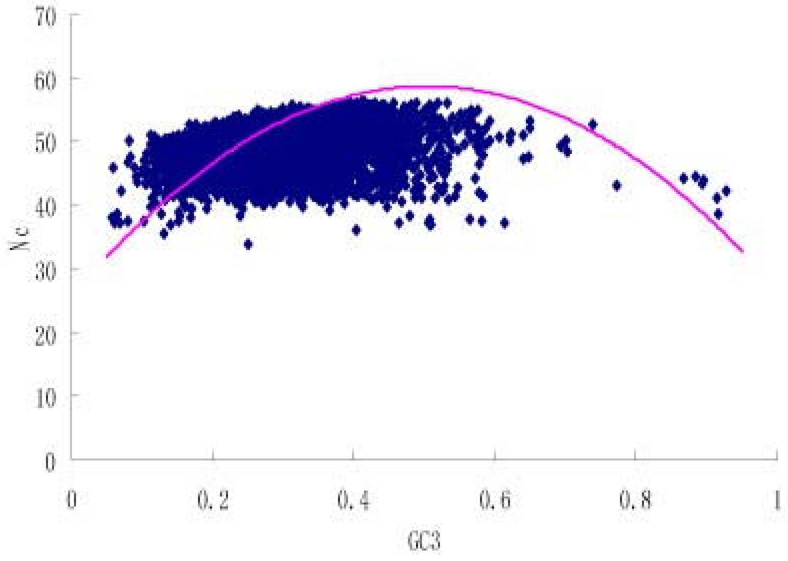

File S4 A plot of Nc versus GC3 (Nc-plot) for *D. antiqua* ORFs. The pink-dotted curve represents the expected curve between GC3 and Nc under random codon usage. A blue dot each indicates corresponding ORFs of each unigene.

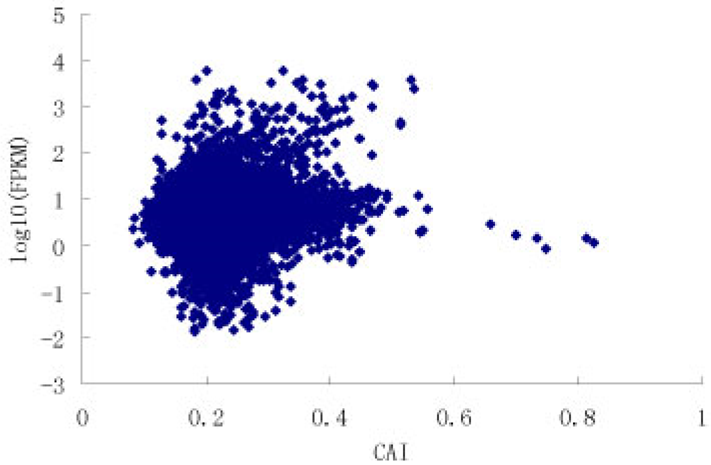

File S5 Relationship between CAI (Codon Adaptation Index) and expression level (Log10 (FPKM)) of all *D. antiqua* transcriptome unigenes.

File S6

SSR identification of *D. antiqua* unigenes.

File S6 is available for download as an Excel file at http://www.g3journal.org/lookup/suppl/doi:10.1534/g3.113.009779/-/DC1
