# Supplementary figures and images for: The de novo Transcriptome and Its Analysis in the Worldwide Vegetable Pest, Delia antiqua (Diptera: Anthomyiidae)
Source: G3 (Bethesda). 2014 Mar 10;4(5):851–9. doi: 10.1534/g3.113.009779 (PMC4025484; doi:10.1534/g3.113.009779)

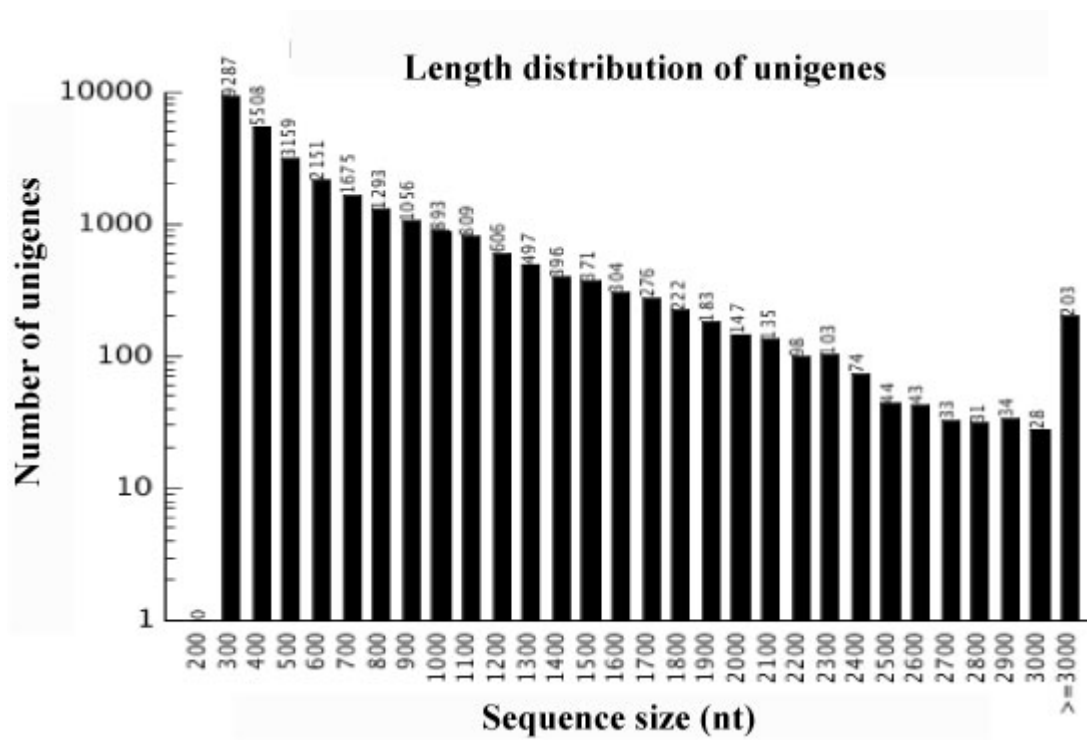

**File S1** Length distribution of unigenes in *D. antiqua*.

Supplement: Supporting Information [file supp_g3.113.009779_FileS1.pdf]
