# Supplementary material for: The de novo Transcriptome and Its Analysis in the Worldwide Vegetable Pest, Delia antiqua (Diptera: Anthomyiidae)
Source: G3 (Bethesda). 2014 Mar 10;4(5):851–9. doi: 10.1534/g3.113.009779 (PMC4025484; doi:10.1534/g3.113.009779)
Supplement: Supporting Information [file supp_g3.113.009779_FileS2.pdf]

**File S2 Total codon usage and codon usage bias in *De. antiqua* transcriptome.** A total of 20578 ORF containing 3913673 codons in the *De. antiqua* transcriptome were used in the calculation of codon usage bias measured by RSCU.

| Codon | Amino Acid | Total codon counts | RSCU | Codon | Amino Acid | Total codon counts | RSCU |
|---|---|---|---|---|---|---|---|
| **CGU** | **R** | 81490 | **2.037** | **UCC** | **S** | 43072 | 0.737 |
| **UUA** | **L** | 118878 | **1.808** | **GGA** | **G** | 44472 | 0.733 |
| **GGU** | **G** | 111980 | **1.623** | **CGC** | **R** | 27367 | 0.686 |
| **UUG** | **L** | 104910 | **1.477** | **GUG** | **V** | 43091 | 0.657 |
| **GAU** | **D** | 172863 | **1.342** | **UCG** | **S** | 39066 | 0.644 |
| **AAU** | **N** | 177112 | **1.333** | **CUA** | **L** | 37341 | 0.569 |
| **GCU** | **A** | 105216 | **1.299** | **UGG** | **W** | 38282 | 0.549 |
| **GAA** | **E** | 202421 | 1.286 | **CUU** | **L** | 34950 | 0.532 |
| **CAA** | **Q** | 147888 | 1.271 | **CGA** | **R** | 18777 | 0.525 |
| **GUU** | **V** | 83193 | 1.247 | **GUC** | **V** | 30508 | 0.445 |
| **AAA** | **K** | 195467 | 1.234 | **UUC** | **F** | 43668 | 0.435 |
| **UAU** | **Y** | 99910 | 1.223 | **AGC** | **S** | 25606 | 0.430 |
| **ACA** | **T** | 87752 | 1.199 | **AAG** | **K** | 72085 | 0.417 |
| **CCA** | **P** | 73462 | 1.188 | **CUG** | **L** | 26604 | 0.393 |
| **CAU** | **H** | 75842 | 1.159 | **CCG** | **P** | 22061 | 0.384 |
| **UUU** | **F** | 107042 | 1.159 | **UGC** | **C** | 21035 | 0.382 |
| **UCA** | **S** | 69422 | 1.143 | **ACG** | **T** | 25727 | 0.368 |
| **AGA** | **R** | 41095 | 1.142 | **CAG** | **Q** | 44046 | 0.363 |
| **AGU** | **S** | 64397 | 1.112 | **CAC** | **H** | 23232 | 0.358 |
| **AUU** | **I** | 104368 | 1.112 | **GAG** | **E** | 55560 | 0.351 |
| **AUA** | **I** | 89316 | 1.024 | **UAC** | **Y** | 29080 | 0.348 |
| **ACU** | **T** | 73298 | 1.014 | **AUC** | **I** | 31938 | 0.348 |
| **UCU** | **S** | 59447 | 0.971 | **AAC** | **N** | 46335 | **0.328** |
| **GCC** | **A** | 75178 | 0.970 | **GAC** | **D** | 37400 | **0.287** |
| **GUA** | **V** | 62064 | 0.961 | **AGG** | **R** | 9837 | **0.282** |
| **UGU** | **C** | 52258 | 0.950 | **GCG** | **A** | 18599 | **0.267** |
| **CCC** | **P** | 53246 | 0.912 | **CUC** | **L** | 16359 | **0.244** |
| **GCA** | **A** | 57320 | 0.798 | **CGG** | **R** | 7571 | **0.206** |
| **CCU** | **P** | 48604 | 0.795 | **GGG** | **G** | 8038 | **0.140** |
| **GGC** | **G** | 51205 | 0.778 | **UAA** | ***** | 685 | 0.029 |
| **ACC** | **T** | 54346 | 0.753 | **UGA** | ***** | 679 | 0.023 |
| **AUG** | **M** | 89348 | 0.744 | **UAG** | ***** | 264 | 0.011 |
