# Supplementary material for: The de novo Transcriptome and Its Analysis in the Worldwide Vegetable Pest, Delia antiqua (Diptera: Anthomyiidae)
Source: G3 (Bethesda). 2014 Mar 10;4(5):851–9. doi: 10.1534/g3.113.009779 (PMC4025484; doi:10.1534/g3.113.009779)
Supplement: Supporting Information [file supp_g3.113.009779_FileS4.pdf]

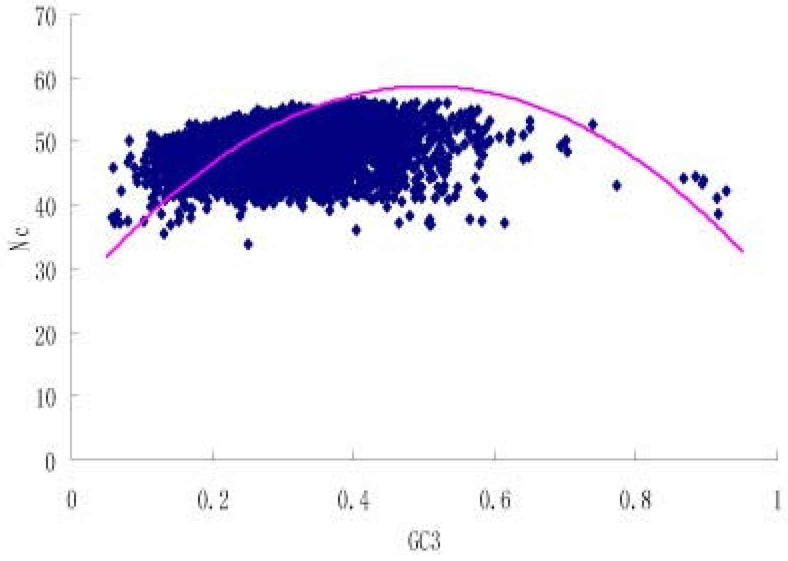

File S4 A plot of Nc versus GC3 (Nc-plot) for *D. antiqua* ORFs. The pink-dotted curve represents the expected curve between GC3 and Nc under random codon usage. A blue dot each indicates corresponding ORFs of each unigene.
