# Supplementary material for: The de novo Transcriptome and Its Analysis in the Worldwide Vegetable Pest, Delia antiqua (Diptera: Anthomyiidae)
Source: G3 (Bethesda). 2014 Mar 10;4(5):851–9. doi: 10.1534/g3.113.009779 (PMC4025484; doi:10.1534/g3.113.009779)
Supplement: Supporting Information [file supp_g3.113.009779_FileS5.pdf]

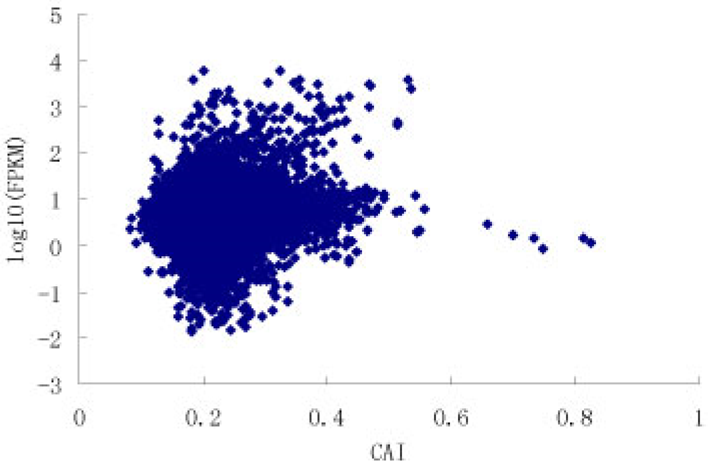

File S5 Relationship between CAI (Codon Adaptation Index) and expression level (Log10 (FPKM)) of all *D. antiqua* transcriptome unigenes.
